# OSluca: An Interactive Web Server to Evaluate Prognostic Biomarkers for Lung Cancer

**DOI:** 10.3389/fgene.2020.00420

**Published:** 2020-05-26

**Authors:** Zhongyi Yan, Qiang Wang, Zhendong Lu, Xiaoxiao Sun, Pengfei Song, Yifang Dang, Longxiang Xie, Lu Zhang, Yongqiang Li, Wan Zhu, Tiantian Xie, Jing Ma, Yijie Zhang, Xiangqian Guo

**Affiliations:** ^1^Department of Predictive Medicine, Institute of Biomedical Informatics, Cell Signal Transduction Laboratory, Bioinformatics Center, Henan Provincial Engineering Center for Tumor Molecular Medicine, School of Software, School of Basic Medical Sciences, Henan University, Kaifeng, China; ^2^Department of Anesthesia, Stanford University, Stanford, CA, United States; ^3^Department of Respiratory and Critical Care Medicine, Huaihe Hospital of Henan University, Kaifeng, China

**Keywords:** survival, lung cancer, biomarker, prognosis, OSluca

## Abstract

Lung cancer is the principal cause of leading cancer-related incidence and mortality in the world. Various studies have excavated the potential prognostic biomarkers for cancer patients based on gene expression profiles. However, most of these reported biomarkers lack independent validation in multiple cohorts. Herein, we collected 35 datasets with long-term follow-up clinical information from TCGA (2 cohorts), GEO (32 cohorts), and Roepman study (1 cohort), and developed a web server named OSluca (Online consensus Survival for Lung Cancer) to assess the prognostic value of genes in lung cancer. The input of OSluca is an official gene symbol, and the output web page of OSluca displays the survival analysis summary with a forest plot and a survival table from Cox proportional regression in each cohort and combined cohorts. To test the performance of OSluca, 104 previously reported prognostic biomarkers in lung carcinoma were evaluated in OSluca. In conclusion, OSluca is a highly valuable and interactive prognostic web server for lung cancer. It can be accessed at http:// bioinfo.henu.edu.cn/LUCA/LUCAList.jsp.

## Introduction

Lung cancer (LUCA) is an aggressive disease with leading mortality and incidence in the world. Based on histology, there are two types of LUCA, including non-small cell lung cancer (NSCLC), which accounts for 80% of LUCA and small cell lung cancer (SCLC), which accounts for approximately 20% of LUCA (Raponi et al., 2006; Bray et al., 2018). NSCLC can be further sub-divided into four subtypes, including adenocarcinoma, squamous cell carcinoma, large cell carcinoma, and bronchioloalveolar carcinoma (Ramalingam et al., 2011). Classical histological subtypes indeed play a dominant role in treatment and prognosis of lung cancer. Recently, reclassification of lung cancer based on tumor biomarkers improves lung cancer therapy (Beer et al., 2002; Hoadley et al., 2018).

Many studies have demonstrated that using clinical-association-prognostic biomarkers can assist the characterization of cancer subtypes and provide new insights of cancer recurrence and patients response to more precise therapies (Meyerson and Carbone, 2005; Bild et al., 2006; Raponi et al., 2006). It is worth noting that numerous single- or multi-prognostic biomarkers have been identified using high-throughput profiling methods (Raponi et al., 2006). By mining a mass of these profiling data deposited in public database, meta-analysis has exploited potential prognostic genes, such as *KRT8* (Xie et al., 2019a). However, for biologists and clinicians, it is technically difficult to analyze these massive public data to screen and develop prognostic biomarkers. Previously, we have built several web servers of prognostic biomarker analysis for breast cancer, esophageal carcinoma, etc. (Wang et al., 2019a,b,c, 2020; Xie et al., 2019b,c; Yan et al., 2019; Zhang et al., 2019, 2020; Dong et al., 2020). In this current study, we have integrated bulky RNA expression profiles of lung cancer with clinical survival information, mainly from TCGA (The Cancer Genome Atlas) and GEO (the Gene Expression Omnibus) databases, and built a prognostic analysis web server named OSluca (Online consensus Survival for Lung Cancer) to analyze and evaluate prognostic potency of gene in 35 independent lung cancer cohorts.

## Materials and Methods

### Collection of Lung Cancer Datasets

The lung cancer cohorts for OSluca with expression profiling and clinical follow-up data were collected from PubMed, TCGA,^1^ and GEO^2^ by searching the keywords: “lung” AND “cancer” AND “survival” (Table 1). The dataset for each cohort that met these following criteria will be included in OSluca: (1) have RNA sequencing or gene microarray data; (2) have complete follow-up data, such as overall survival and status (Liu et al., 2018); (3) all the data were specific for lung cancer, not from secondary or metastatic lung tumor from other types of tumors; (4) the cohort size is no less than 30 cases. The primary clinical pathological characteristics of lung cancer patients are listed in Table 1.

**TABLE 1:** Summary of clinical characteristics of lung cancer cohorts in Online Consensus Survival for Lung Cancer (OSluca).

	**NSCLC**					**SCLC (*N* = 223)**	**#NA (*N* = 85)**
	**NSCLC, Total (*N* = 4937)**	**AD (*N* = 3345)**	**SCC (*N* = 1381)**	**LCC (*N* = 197)**	**NOS (*N* = 194)**		
Age, year	64 (13–91)	64 (13–90)	66 (39–83)	63 (39–81)	62 (22–80)	64 (40–83)	58 (15–82)
Gender							
Male, %	52.6	46.9	68.3	77.2	12.9	58.1	50
Female, %	38.8	47.7	23.7	18.1	12.4	41.9	50
#NA, %	8.6	5.4	8.0	4.7	73.7	0	0
Stage*							
I, *n*	2301	1,653	567	66	28	10	9
II, *n*	889	500	347	27	15	5	4
III, *n*	595	366	199	18	12	2	3
IV, *n*	101	73	13	2	13	0	0
T stage 1/2/3/4/#NA	646/1074/230/103/2884	468/663/102/49/2063	155/362/109/39/716	20/44/17/9/107	3/5/2/6/178	11/13/5/4/190	28/20/10/6/21
N Stage 0/1/2/3/#NA	1638/495/280/21	1038/254/198/5/1859	549/218/70/7/537	48/20/17/5/107	3/3/4/4/180	14/4/12/6/187	33/25/5/1/21
M stage 0/1/#NA	1685/42/3210	853/26/2466	740/8/633	82/2/113	10/6/178	33/4/186	63/2/20
Smoking/non-smoking/#NA	1839/262/2836	1112/256/1977	618/3/760	40/1/156	9/2/183	18/1/204	9/8/68
OS, mo	46 (0.03–256)	48 (0.03–242)	41 (0.03–256)	46 (0.1–216)	38 (0.5–208)	51 (2–211)	68 (2–244)
DSS, mo	42 (0.03–256)	43 (0.19–242)	41 (0.03–256)	45 (1–216)	36 (6–76)	24 (2–140)	69 (2–244)
DFI, mo	33 (0.16–242)	32 (0.6–242)	34 (0.16–159)	–	–	–	–
PFI, mo	33 (0.03–242)	36 (0.03–242)	30 (0.03–180)	53 (1.8–164)	4 (0.23–54)	–	30 (2–73)

NSCLC, non-small cell lung cancer; SCLC, small cell lung cancer; AD, adenocarcinoma; SCC, squamous cell carcinoma; LCC, large cell cancer; NOS, NSCLC, not otherwise specified; F, female; M, male; n, number; mo, months; OS, overall survival; DSS, disease-specific survival; DFI, disease-free interval; PFI, progression-free interval or recurrence free survival. *The stage only counts stages of lung cancer patients described in the original datasets; #NA, data lost or unknown.

### Construction of OSluca Web Server

Online consensus Survival for Lung Cancer is built in a tomcat server as previously described with minor modifications (Wang et al., 2019b, c; Xie et al., 2019b, c; Yan et al., 2019; Zhang et al., 2019). Briefly, front-end application was used for inputting query and displaying the results. Java and R package were used to analyze request and output the results. In addition, profiles and clinical information were stored in the SQL Server database. The prognostic significance of inputted gene is determined by analyzing the association of gene expression and survival time using the R package “survival.” In addition, a genome-wide pre-calculation of Cox proportional regression for all the human genes were performed as well, and the home page of OSluca could display the survival analysis summary with a forest plot and a table of Cox proportional regression result for inputted gene in all cohorts with *P*-value and HR [(95% confidence interval (CI)] with the built-in upper 25% cutoff. The R package “forestplot” was used to produce the forest plot for inputted gene in OSluca web server.

### Validation of Previously Reported Prognostic Biomarkers of Lung Cancer in OSluca

Keywords including “lung cancer,” “survival,” “biomarker,” and “prognosis” were used to search biomarkers of lung cancer in NCBI PubMed. We finally obtained 104 prognostic biomarkers using the following criteria (Table 2): (1) immunohistochemistry (IHC) or qRT-PCR (qPCR) detection of biomarkers in primary cancer tissue; (2) a significant association between biomarker and survival; (3) the sample size must be above 50 cases; (4) the study was published in the English for full access.

**TABLE 2:** Clinico-pathological traits of lung cancer cohorts.

**Datasets**	**Cohorts**	**Platform**	**Histological type**	**Survival**	**Samples**	**References**
Rockville	GSE102287	GPL570	AD/SCC/NOS	OS	32	Mitchell et al., 2017
Heidelberg	GSE10245	GPL570	AD/SCC	OS	58	Kuner et al., 2009
Koto-ku	GSE1037	GPL962	AD/SCC/SCLC	OS	61	Jones et al., 2004
Basel	GSE11117	GPL6650	AD/SCC/NOS	OS	41	Baty et al., 2010
Nagoya	GSE11969	GPL7015	AD/SCC/LCC	OS	149	Takeuchi et al., 2006
Groningen	GSE12428	GPL1708	SCC	OS	34	Boelens et al., 2009
Nagoya	GSE13213	GPL6480	AD	OS	117	Tomida et al., 2009
Toronto	GSE14814	GPL96	AD/SCC/NOS	OS/DSS	133	Zhu et al., 2010
Chapel Hill	GSE17710	GPL9053	SCC	OS/PFI	56	Wilkerson et al., 2010
Rotterdam	GSE19188	GPL570	AD/SCC/LCC	OS	82	Hou et al., 2010
Chapel Hill	GSE26939	GPL9053	AD	OS	116	Wilkerson et al., 2012
Dallas	GSE29013	GPL570	AD/SCC	OS/PFI	55	Xie et al., 2011
Lund	GSE29066	GPL6947	AD/SCC/SCLC	OS	68	Staaf et al., 2012, 2013
La Tronche	GSE30219	GPL570	AD/SCC/SCLC/LCC	OS/DFS	293	Rousseaux et al., 2013
Chuo-ku	GSE31210	GPL570	AD	OS/PFI	226	Okayama et al., 2012
Durham	GSE3141	GPL570	AD/SCC	OS	111	Bild et al., 2006
Dallas	GSE31908	GPL96/97	AD	OS	30	NA
Houston	GSE33072	GPL6244	AD/SCC	PFI	66	Byers et al., 2013
Uppsala	GSE37745	GPL570	AD/SCC/LCC	PFI	196	Botling et al., 2013
Dallas	GSE41271	GPL6884	AD/SCC/LCC	OS/PFI	275	Sato et al., 2013
San Diego	GSE4573	GPL96	SCC	OS	130	Raponi et al., 2006
Nagoya	GSE4716	GPL3696/3694	AD/SCC/LCC	OS	50	Tomida et al., 2004
Toronto	GSE50081	GPL570	AD/SCC/LCC	OS/DFS	181	Der et al., 2014
Brisbane	GSE5123	GPL3877	SCC	OS	51	Larsen et al., 2007b
Brisbane	GSE5828	GPL3877	SCC	OS	59	Larsen et al., 2007a
Brisbane	GSE5843	GPL3877	AD	OS	48	Larsen et al., 2007c
St. Louis	GSE6253	GPL8300	AD/SCC/NOS	DSS	34	Lu et al., 2006
Bethesda	GSE63459	GPL6883	AD	OS	33	Robles et al., 2015
Stanford	GSE67639	GPL570	AD/SCC/NOS	OS	1106	Gentles et al., 2015
Rockville	GSE68465	GPL96	AD	OS/PFI	442/363	Shedden et al., 2008
Rockville	GSE68571	GPL80	AD	OS	86	Beer et al., 2002
Seoul	GSE8894	GPL570	AD/SCC	PFI	138	Lee et al., 2008
NIH and NHGRI	TCGA	DCC	AD	OS/DSS/DFI/PFI	513/478/306/513	The Cancer Genome Atlas Research Network, 2014; Liu et al., 2018
NIH and NHGRI	TCGA	DCC	SCC	OS/DSS/DFI/PFI	498/452/303/499	Hammerman et al., 2012; The Cancer Genome Atlas Research Network, 2012; Liu et al., 2018
Reopman	Roepman		AD/SCC/LCC/NOS	OS	172	Roepman et al., 2009

*NSCLC, non-small cell lung cancer; SCLC, small cell lung cancer; AD, adenocarcinoma; SCC, squamous cell carcinoma; LCC, large cell carcinoma; NOS, not otherwise specified; OS, overall survival; DSS, disease-specific survival; DFI, disease-free interval; PFI, progression-free interval.*

### Statistical Analysis

The association of lung cancer clinical factors and survival outcomes was analyzed by GraphPad Prism 8.0 software. The Cox proportional hazards regression and Kaplan Meier plot functions from R package “survival” were used in the OSluca to determine the association between gene expression and survival. The *P* ≤ 0.05 was considered statistically significant.

## Results

### Clinical Characteristics of Lung Cancer Patients in OSluca

**FIGURE 1:**
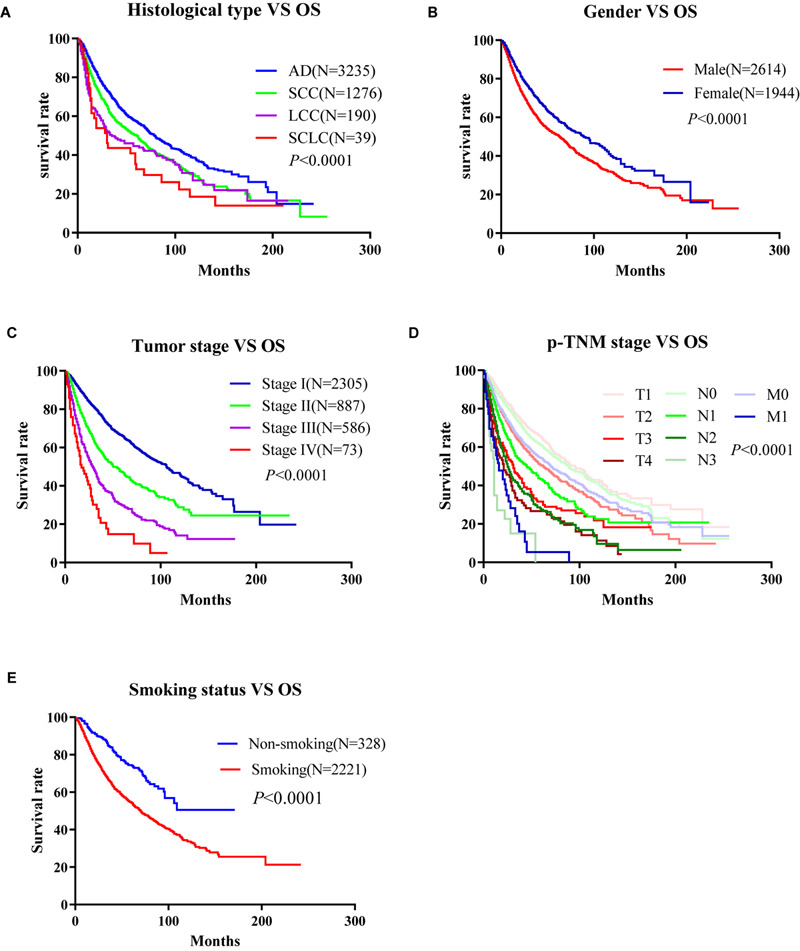
Correlation between the clinico-pathologic characteristics and overall survival of lung cancer in Online Consensus Survival for Lung Cancer (OSluca). **(A)** Correlation between histological types and OS. **(B)** Correlation between gender and OS. **(C)** Correlation between tumor stages and OS. **(D)** Correlation between p-TNM stages and OS. **(E)** Correlation between smoking status and OS. OS, overall survival; AD, adenocarcinoma; SCC, squamous cell carcinoma; LCC, large cell cancer.

To develop an online survival web server for lung cancer, we collected 35 published high-throughput profiling datasets of lung cancer with long-term follow-up information (2 TCGA datasets, 32 GEO datasets, and 1 Roepman dataset). TCGA comprises 513 lung adenocarcinoma cases and 499 squamous cell carcinoma cases (Tables 1, 2). GEO cohorts and Roepman cohort had more than 4,000 samples and 172 samples, respectively, as shown in Table 2. 4,901 patients have OS (overall survival) data; 2,176 patients have DSS (disease-specific survival) data; and 2,075 patients have PFI (progression-free interval or recurrence-free survival) data, while 608 patients have DFI (disease-free interval) data. The results showed that the patients with lung adenocarcinoma significantly survive longer than those of other histological lung cancer, and small cell lung cancer is associated with the worst prognosis compared to other types of lung cancer (Figure 1A). Moreover, other clinical characteristics can also prominently affect patients’ prognosis, such as gender (*P* < 0.0001), stage (*P* < 0.0001), p-TNM stage (*P* < 0.0001), and smoking status (*P* < 0.0001) (Figures 1B–E). Besides, these risk factors can influence other survival endpoints, such as PFI (data not shown). These results are in accordance with previous researches (Mao et al., 2016; Bray et al., 2018).

### Construction and Usage of Prognostic Web Server OSluca

**FIGURE 2:**
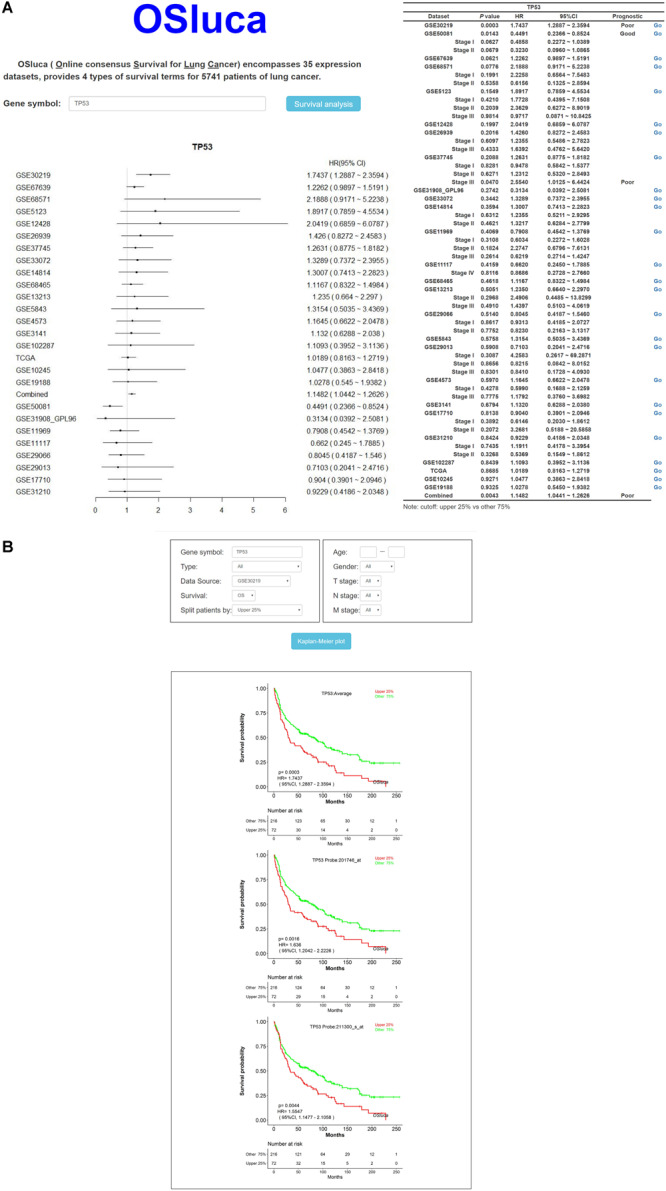
The output home page and KM output web subpage in OSluca for lung cancer. **(A)** Home page of OSluca with *TP53* gene survival analysis, containing prognostic meta-analysis of a forest plot and a survival table. **(B)** KM plots of *TP53* gene in the GSE30219 cohort. Note: the cutoff value is the upper 25% vs. other 75%. The “Combined” in forest plot and survival table means the overall prognostic significance of inputted gene in a pooling cohort with all the datasets. TP53, tumor protein p53.

Online consensus Survival for Lung Cancer includes a set of optional clinico-pathological factors, such as age, sex, histological type, grade, smoking status, and so on. Four survival endpoints can be selected basing on original patient outcomes, containing OS, DSS, DFI, and PFI (Liu et al., 2018). In order to make the user clearly see the prognostic effect of interested gene, a meta-analysis is to summarize the prognostic value for each gene on the home page of OSluca. Briefly, after the user types the official gene symbol into the input box on the home page, OSluca will display the survival analysis summary with a forest plot and a table from Cox proportional regression in each cohort and combined cohorts (combining all the datasets together). Take the tumor suppressor gene *TP53* (tumor protein p53) as an example and type “TP53” into the gene symbol box and click on “Survival analysis” (Figure 2A, left). The meta-analysis results with a forest plot and a survival table for the *TP53* gene, will display the *P*-value and HR with 95% CI of each cohort and the combined cohorts (Figure 2A, right). Then, the user can easily obtain KM plots of separate cohorts such as GSE30219 dataset by clicking on the “Go” button in the survival table (Figure 2B). In addition, it is also available to use a subgroup of certain cohort to obtain specific prognostic information with selectable risk factors, such as cutoff value, histological type, grade, etc. Briefly, OSluca can output survival rates displaying a forest plot and a survival table with KM plot and *P*-value to measure the association between the investigated gene and survival rate.

### Validation of Previously Reported Lung Cancer Prognostic Biomarkers in OSluca

**FIGURE 3:**
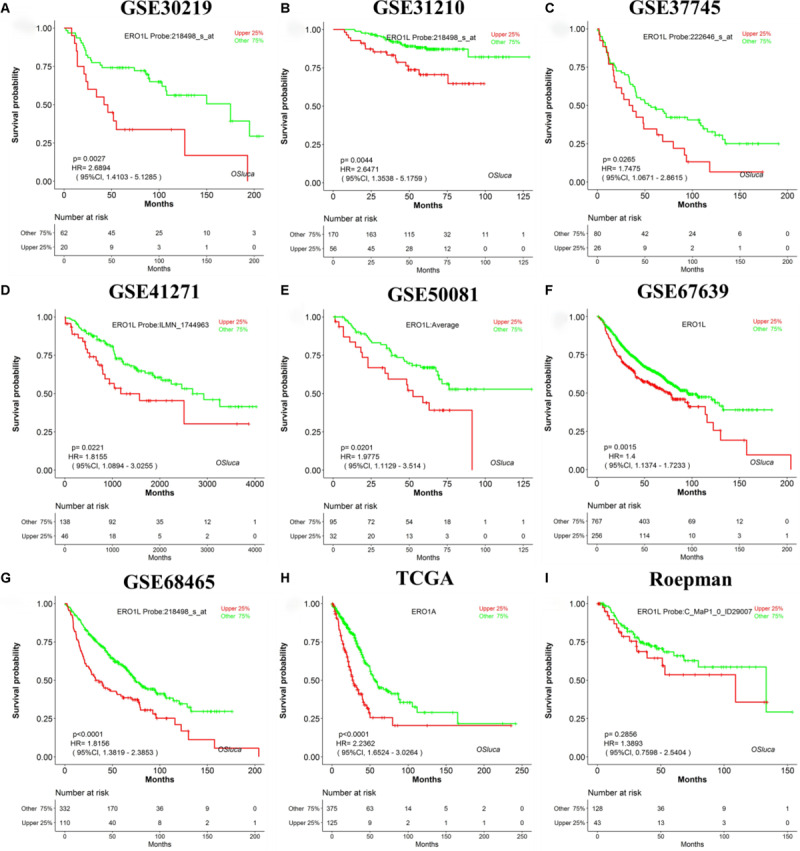
Validation of a previously reported biomarker *ERO1L* in OSluca. Overexpression of *ERO1L* in tumor tissue is suggested as a worse survival biomarker in lung adenocarcinoma. **(A)** Overall survival (OS) of *ERO1L* gene in GSE30219 cohort. **(B)** OS in GSE31210 cohort. **(C)** OS in GSE37745 cohort. **(D)** OS in GSE41271 cohort. **(E)** OS in GSE50081 cohort. **(F)** OS in GSE67639 cohort. **(G)** OS in GSE68465 cohort. **(H)** OS in TCGA in lung adenocarcinoma. **(I)** OS in Roepman cohort. The histological type of all the above cohorts is lung adenocarcinoma. ERO1L, ERO1-like protein alpha (also named ERO1A).

A search for lung cancer biomarkers was performed using a set of keywords in NCBI PubMed, including “lung cancer,” “survival,” “biomarker,” and “prognosis.” In total, we collected 104 published lung cancer prognostic biomarkers verified by IHC or qPCR (Supplementary Table S1) to evaluate the performance of OSluca. For example, Hsu et al. reported that *ERO1L* (ERO1-like protein alpha, also named *ERO1A*) is significantly overexpressed in tumor tissue and could be as a poor prognostic biomarker for lung adenocarcinoma (Hsu et al., 2016). The prognostic analysis of *ERO1L* in OSluca showed that high expression of *ERO1L* gene is significantly associated with poor outcome in eight out of nine cohorts (Top 9 cohorts, the sample size above 150 cases) (Figures 3A–H), except the Roepman dataset (Figure 3I). Next, each published biomarker was investigated in the Top 9 cohorts in OSluca, and the results showed that approximately 66% of biomarkers (69/104) were consistent with original published findings (Supplementary Table S1). Meanwhile, OSluca can be used to perform the outcome meta-analysis of the interested gene that showed that 14% (14/104) (Supplementary Table S1) of published prognostic genes have the similar prognostic values in one or multiple OSluca cohorts as reported in the literature, but these genes also showed the opposite outcomes in some other cohorts from OSluca. These genes need further investigations, such as the *DDIT3* gene (Supplementary Figure S2 and Supplementary Table S1). In contrast, there are some prognostic biomarkers, which have been shown different outcomes between OSluca and previous findings. A total of 9% of the published prognostic genes showed opposite outcome results between OSluca and literatures (9/104) (see Supplementary Table S1), suggesting that these genes need further validation. For example, the transcription factor *KLF15* (Krüppel-like factor 15) had been proven to be higher in tumor tissue than that of adjacent non-tumor tissue and played an important role in promoting proliferation and carcinoma diversification in lung adenocarcinoma, associated with poor prognostic outcome (Gao et al., 2017). It was not anticipated that the patients with high expression of *KLF15* have better survival than those with low expression (Supplementary Table S1 and Supplementary Figure S1). The OSluca result for the *KLF15* gene was consistent with other prognostic analysis tools (Gyõrffy et al., 2013; Anaya, 2016), such as the KM plotter [*P* < 0.001, HR (95% CI) = 0.4 (0.28–0.58)]. In addition, the remaining 12 of 104 previously published prognostic biomarkers (11%) were not significant for prognostic analysis in the Top 9 cohorts in OSluca, but 8 of them (8/12) are significant in one or multiple datasets other than the Top 9 cohorts in OSluca (data not shown). All in all, the OSluca server is an interactive and free web server for researchers to develop potential prognostic biomarkers for lung cancer.

## Discussion

Owing to tumor molecular heterogeneity, the prognosis of lung cancer patients is variable and difficult to predict. The prognosis of patients suffering from lung cancer had been demonstrated to be highly dependent on clinical factors of the patient, such as histological type, smoking status, and so on. However, it is also an imperative need to exploit novel prognostic biomarkers for determining the risk of cancerous lesions and predicting lung cancer patient outcomes by all available means, especially by high-throughput sequencing technologies. However, one major challenge to non-bioinformatics researchers is how to integrate the high-dimension profiling datasets of lung cancer and discover new biomarkers to potentially guide prognostic stratification. Previous studies had revealed that the online prognostic web servers of cancer (Elfilali et al., 2006; Mizuno et al., 2009; Goswami and Nakshatri, 2013; Gyõrffy et al., 2013; Tang et al., 2017) could substantially help researchers to discover potential biomarkers (Zheng et al., 2020). Herein, we developed a free web server OSluca to assess the prognostic value of the interesting gene in multiple cohorts of lung cancers. In OSluca, all the lung cancer cases are originated from the organ lung, not the second cancer from other cancers or organs. As a result, the prognostic specificity is only for lung cancer. Nevertheless, its prognostic significance in other types of cancers is also worth to be determined. To access the repeatability of previously reported prognostic biomarkers in OSluca, we collected 104 previously published prognostic biomarkers of lung cancer identified by qPCR or IHC, and tested their prognostic significance in OSluca. The testing results showed that most of the biomarkers were verified in OSluca and were confirmed for the published findings. Nevertheless, some genes showed different prognostic outcomes compared to previous literatures.

The advantage of OSluca over other online prognostic web servers is that the size of lung cancer samples in OSluca is large, and tens of independent cohorts are available, which is extremely valuable for the identification and validation of cancer prognostic biomarkers, since the most important part for the biomarker development is independent validation across different datasets/cohorts. The limitation of the current study is that OSluca can only test a single gene for outcome analysis. In summary, OSluca is a free web server for non-bioinformatics researchers to study potential lung cancer prognostic biomarkers, accessed at http://bioinfo.henu.edu.cn/LUCA/LUCAList.jsp.

## Data Availability Statement

The datasets generated for this study can be found in the TCGA, NCBI GEO, and Roepman dataset.

## Author Contributions

XG: research design. QW and XG: establish OSluca web server. ZY, ZL, and XS: deal with RNA sequencing with clinical data of lung cancer. ZY, LX, XS, LZ, YL, and XG: draft of the manuscript. YD, XS, LZ, PS, YL, TX, and JM: collect previously reported biomarkers of lung cancer. ZY, LX, LZ, WZ, YZ, and XG: critical revision of the manuscript.

## Conflict of Interest

The authors declare that the research was conducted in the absence of any commercial or financial relationships that could be construed as a potential conflict of interest.

## Footnotes

1 https://cancergenome.nih.gov/

2 www.ncbi.nlm.nih.gov/geo/

## References

[B1] Anaya J. (2016). OncoLnc: linking TCGA survival data to mRNAs, miRNAs, and lncRNAs. *Peerj Comput. Sci.* 2:e67.

[B2] Baty F. Facompre M. Kaiser S. Schumacher M. Pless M. Bubendorf L. (2010). Gene profiling of clinical routine biopsies and prediction of survival in non-small cell lung cancer. *Am. J. Respir. Crit. Care Med.* 181 181–188. 10.1164/rccm.200812-1807OC 19833826

[B3] Beer D. G. Kardia S. L. Huang C. C. Giordano T. J. Levin A. M. Misek D. E. (2002). Gene-expression profiles predict survival of patients with lung adenocarcinoma. *Nat. Med.* 8 816–824. 10.1038/nm733 12118244

[B4] Bild A. H. Yao G. Chang J. T. Wang Q. Potti A. Chasse D. (2006). Oncogenic pathway signatures in human cancers as a guide to targeted therapies. *Nature* 439 353–357. 10.1038/nature04296 16273092

[B5] Boelens M. C. van den Berg A. Fehrmann R. S. Geerlings M. de Jong W. K. te Meerman G. J. (2009). Current smoking-specific gene expression signature in normal bronchial epithelium is enhanced in squamous cell lung cancer. *J. Pathol.* 218 182–191. 10.1002/path.2520 19334046

[B6] Botling J. Edlund K. Lohr M. Hellwig B. Holmberg L. Lambe M. (2013). Biomarker discovery in Non–small cell lung cancer: integrating gene expression profiling, meta-analysis, and tissue microarray validation. *Clin. Cancer Res.* 19 194–204. 10.1158/1078-0432.ccr-12-1139 23032747

[B7] Bray F. Ferlay J. Soerjomataram I. Siegel R. L. Torre L. A. Jemal A. (2018). Global cancer statistics 2018: GLOBOCAN estimates of incidence and mortality worldwide for 36 cancers in 185 countries. *Cancer J. Clin.* 68 394–424. 10.3322/caac.21492 30207593

[B8] Byers L. A. Diao L. Wang J. Saintigny P. Girard L. Peyton M. (2013). An epithelial-mesenchymal transition gene signature predicts resistance to EGFR and PI3K inhibitors and identifies Axl as a therapeutic target for overcoming EGFR inhibitor resistance. *Clin. Cancer Res.* 19 279–290. 10.1158/1078-0432.CCR-12-1558 23091115 PMC3567921

[B9] Der S. D. Sykes J. Pintilie M. Zhu C.-Q. Strumpf D. Liu N. (2014). Validation of a histology-independent prognostic gene signature for early-stage, non–small-cell lung cancer including stage IA patients. *J. Thorac. Oncol.* 9 59–64. 10.1097/JTO.0000000000000042 24305008

[B10] Dong H. Wang Q. Zhang G. Li N. Yang M. An Y. (2020). OSdlbcl: an online consensus survival analysis web server based on gene expression profiles of diffuse large B-cell lymphoma. *Cancer Med.* 9 1790–1797. 10.1002/cam4.2829 31918459 PMC7050097

[B11] Elfilali A. Lair S. Verbeke C. La Rosa P. Radvanyi F. Barillot E. (2006). ITTACA: a new database for integrated tumor transcriptome array and clinical data analysis. *Nucleic Acids Res.* 34 D613–D616. 10.1093/nar/gkj022 16381943 PMC1347385

[B12] Gao L. Qiu H. Liu J. Ma Y. Feng J. Qian L. (2017). KLF15 promotes the proliferation and metastasis of lung adenocarcinoma cells and has potential as a cancer prognostic marker. *Oncotarget* 8 109952–109961. 10.18632/oncotarget.21972 29299121 PMC5746356

[B13] Gentles A. J. Bratman S. V. Lee L. J. Harris J. P. Feng W. Nair R. V. (2015). Integrating tumor and stromal gene expression signatures with clinical indices for survival stratification of early-stage Non-small cell lung cancer. *J. Natl. Inst.* 107:djv211 10.1093/jnci/djv211 PMC6090873 26286589

[B14] Goswami C. P. Nakshatri H. (2013). PROGgene: gene expression based survival analysis web application for multiple cancers. *J. Clin. Bioinform.* 3:22 10.1186/2043-9113-3-22 PMC3875898 24165311

[B15] Gyõrffy B. Surowiak P. Budczies J. Lánczky A. (2013). Online survival analysis software to assess the prognostic value of biomarkers using transcriptomic data in non-small-cell lung cancer. *PLoS One* 8:e82241 10.1371/journal.pone.0082241 PMC3867325 24367507

[B16] Hammerman P. S. Lawrence M. S. Voet D. Jing R. Cibulskis K. Sivachenko A. (2012). Comprehensive genomic characterization of squamous cell lung cancers. *Nature* 489 519–525. 10.1038/nature11404 22960745 PMC3466113

[B17] Hoadley K. A. Yau C. Hinoue T. Wolf D. M. Lazar A. J. Drill E. (2018). Cell-of-origin patterns dominate the molecular classification of 10,000 tumors from 33 Types of cancer. *Cell* 173 291.e6–304.e6. 10.1016/j.cell.2018.03.022 29625048 PMC5957518

[B18] Hou J. Aerts J. den Hamer B. van Ijcken W. den Bakker M. Riegman P. (2010). Gene expression-based classification of non-small cell lung carcinomas and survival prediction. *PloS One* 5:e10312 10.1371/journal.pone.0010312 PMC2858668 20421987

[B19] Hsu C.-H. Hsu C.-W. Hsueh C. Wang C.-L. Wu Y.-C. Wu C.-C. (2016). Identification and characterization of potential biomarkers by quantitative tissue proteomics of primary lung adenocarcinoma. *Mol. Cell. Proteomics* 15 2396–2410. 10.1074/mcp.M115.057026 27161446 PMC4937512

[B20] Jones M. H. Virtanen C. Honjoh D. Miyoshi T. Satoh Y. Okumura S. (2004). Two prognostically significant subtypes of high-grade lung neuroendocrine tumours independent of small-cell and large-cell neuroendocrine carcinomas identified by gene expression profiles. *Lancet* 363 775–781. 10.1016/s0140-6736(04)15693-6 15016488

[B21] Kuner R. Muley T. Meister M. Ruschhaupt M. Buness A. Xu E. C. (2009). Global gene expression analysis reveals specific patterns of cell junctions in non-small cell lung cancer subtypes. *Lung Cancer* 63 32–38. 10.1016/j.lungcan.2008.03.033 18486272

[B22] Larsen J. E. Pavey S. J. Bowman R. Yang I. A. Clarke B. E. Colosimo M. L. (2007a). Gene expression of lung squamous cell carcinoma reflects mode of lymph node involvement. *Eur. Respir. J.* 30 21–25. 10.1183/09031936.00161306 17601969

[B23] Larsen J. E. Pavey S. J. Passmore L. H. Bowman R. Clarke B. E. Hayward N. K. (2007b). Expression profiling defines a recurrence signature in lung squamous cell carcinoma. *Carcinogenesis* 28 760–766. 10.1093/carcin/bgl207 17082175

[B24] Larsen J. E. Pavey S. J. Passmore L. H. Bowman R. V. Hayward N. K. Fong K. M. (2007c). Gene expression signature predicts recurrence in lung adenocarcinoma. *Clin. Cancer Res.* 13 2946–2954. 10.1158/1078-0432.ccr-06-2525 17504995

[B25] Lee E.-S. Son D.-S. Kim S.-H. Lee J. Jo J. Han J. (2008). Prediction of recurrence-free survival in postoperative Non–small cell lung cancer patients by using an integrated model of clinical information and gene expression. *Clin. Cancer Res.* 14 7397–7404. 10.1158/1078-0432.ccr-07-4937 19010856

[B26] Liu J. Lichtenberg T. Hoadley K. A. Poisson L. M. Lazar A. J. Cherniack A. D. (2018). An integrated tcga pan-cancer clinical data resource to drive high-quality survival outcome analytics. *Cell* 173 400.e11–416.e11. 10.1016/j.cell.2018.02.052 29625055 PMC6066282

[B27] Lu Y. Lemon W. Liu P.-Y. Yi Y. Morrison C. Yang P. (2006). A gene expression signature predicts survival of patients with stage I non-small cell lung cancer. *PLoS Med.* 3:e030467 10.1371/journal.pmed.0030467 PMC1716187 17194181

[B28] Mao Y. Yang D. He J. Krasna M. J. (2016). Epidemiology of lung cancer. *Surg. Oncol. Clin. North Am.* 25 439–445. 10.1016/j.soc.2016.02.001 27261907

[B29] Meyerson M. Carbone D. (2005). Genomic and proteomic profiling of lung cancers: lung cancer classification in the age of targeted therapy. *J. Clin. Oncol.* 23 3219–3226. 10.1200/JCO.2005.15.511 15886309

[B30] Mitchell K. A. Zingone A. Toulabi L. Boeckelman J. Ryan B. M. (2017). Comparative transcriptome profiling reveals coding and noncoding RNA differences in NSCLC from african americans and european americans. *Clin. Res.* 23 7412–7425. 10.1158/1078-0432.ccr-17-0527 PMC8171584 29196495

[B31] Mizuno H. Kitada K. Nakai K. Sarai A. (2009). PrognoScan: a new database for meta-analysis of the prognostic value of genes. *BMC Med. Genomics* 2:18 10.1186/1755-8794-2-18 PMC2689870 19393097

[B32] Okayama H. Kohno T. Ishii Y. Shimada Y. Shiraishi K. Iwakawa R. (2012). Identification of genes up-regulated in ALK-positive and EGFR/KRAS/ALK-negative lung adenocarcinomas. *Cancer Res.* 72 100–111. 10.1158/0008-5472.can-11-1403 22080568

[B33] Ramalingam S. S. Owonikoko T. K. Khuri F. R. (2011). Lung cancer: new biological insights and recent therapeutic advances. *Cancer J. Clin.* 61 91–112. 10.3322/caac.20102 21303969

[B34] Raponi M. Zhang Y. Yu J. Chen G. Lee G. Taylor J. M. (2006). Gene expression signatures for predicting prognosis of squamous cell and adenocarcinomas of the lung. *Cancer Res.* 66 7466–7472. 10.1158/0008-5472.can-06-1191 16885343

[B35] Robles A. I. Arai E. Mathé E. A. Okayama H. Schetter A. J. Brown D. (2015). An integrated prognostic classifier for stage I lung adenocarcinoma based on mRNA, microRNA, and DNA methylation biomarkers. *J. Thora.c Oncol.* 10 1037–1048. 10.1097/JTO.0000000000000560 PMC4493931 26134223

[B36] Roepman P. Jassem J. Smit E. F. Muley T. Niklinski J. van de Velde T. (2009). An Immune response enriched 72-gene prognostic profile for early-stage non–small-cell lung cancer. *Clin. Cancer Res.* 15 284–290. 10.1158/1078-0432.ccr-08-1258 19118056

[B37] Rousseaux S. Debernardi A. Jacquiau B. Vitte A.-L. Vesin A. Nagy-Mignotte H. (2013). Ectopic activation of germline and placental genes identifies aggressive metastasis-prone lung cancers. *Sci. Transl. Med.* 5:ra66 10.1126/scitranslmed.3005723 PMC4818008 23698379

[B38] Sato M. Larsen J. E. Lee W. Sun H. Shames D. S. Dalvi M. P. (2013). Human lung epithelial cells progressed to malignancy through specific oncogenic manipulations. *Mol. Cancer Res.* 11 638–650. 10.1158/1541-7786.MCR-12-0634-T 23449933 PMC3687022

[B39] Shedden K. Taylor J. M. G. Enkemann S. A. Tsao M.-S. Yeatman T. J. Gerald W. L. (2008). Gene expression-based survival prediction in lung adenocarcinoma: a multi-site, blinded validation study. *Nat. Med.* 14 822–827. 10.1038/nm.1790 18641660 PMC2667337

[B40] Staaf J. Isaksson S. Karlsson A. Jonsson M. Johansson L. Jonsson P. (2013). Landscape of somatic allelic imbalances and copy number alterations in human lung carcinoma. *Int. J. Cancer* 132 2020–2031. 10.1002/ijc.27879 23023297

[B41] Staaf J. Jönsson G. Jönsson M. Karlsson A. Isaksson S. Salomonsson A. (2012). Relation between smoking history and gene expression profiles in lung adenocarcinomas. *BMC Med. Genomics* 5:22 10.1186/1755-8794-5-22 PMC3447685 22676229

[B42] Takeuchi T. Tomida S. Yatabe Y. Kosaka T. Osada H. Yanagisawa K. (2006). Expression profile–defined classification of lung adenocarcinoma shows close relationship with underlying major genetic changes and clinicopathologic behaviors. *J. Clin. Oncol.* 24 1679–1688. 10.1200/JCO.2005.03.8224 16549822

[B43] Tang Z. Li C. Kang B. Gao G. Li C. Zhang Z. (2017). GEPIA: a web server for cancer and normal gene expression profiling and interactive analyses. *Nucleic Acids Res.* 45 W98–W102. 10.1093/nar/gkx247 28407145 PMC5570223

[B44] The Cancer Genome Atlas Research Network (2012). Comprehensive genomic characterization of squamous cell lung cancers. *Nature* 489 519– 525. 22960745 10.1038/nature11404 PMC3466113

[B45] The Cancer Genome Atlas Research Network (2014). Comprehensive molecular profiling of lung adenocarcinoma. *Nature* 511 543–550. 10.1038/s41586-018-0228-6 25079552 PMC4231481

[B46] Tomida S. Koshikawa K. Yatabe Y. Harano T. Ogura N. Mitsudomi T. (2004). Gene expression-based, individualized outcome prediction for surgically treated lung cancer patients. *Oncogene* 23 5360–5370. 10.1038/sj.onc.1207697 15064725

[B47] Tomida S. Takeuchi T. Shimada Y. Arima C. Matsuo K. Mitsudomi T. (2009). Relapse-related molecular signature in lung adenocarcinomas identifies patients with dismal prognosis. *J. Clin. Oncol.* 27 2793–2799. 10.1200/JCO.2008.19.7053 19414676

[B48] Wang F. Wang Q. Li N. Ge L. Yang M. An Y. (2019a). OSuvm: an interactive online consensus survival tool for uveal melanoma prognosis analysis. *Mol. Carcinog.* 59 56–61. 10.1002/mc.23128 31646691

[B49] Wang F. Wang Q. Li N. Ge L. Yang M. An Y. (2020). OSuvm: an interactive online consensus survival tool for uveal melanoma prognosis analysis. *Mol. Carcinog.* 59 56–61. 31646691 10.1002/mc.23128

[B50] Wang Q. Xie L. Dang Y. Sun X. Xie T. Guo J. (2019b). OSlms: a web server to evaluate the prognostic value of genes in leiomyosarcoma. *Front. Oncol.* 9:190 10.3389/fonc.2019.00190 PMC6449415 30984618

[B51] Wang Q. Zhang L. Yan Z. Xie L. (2019c). OScc: an online survival analysis web server to evaluate the prognostic value of biomarkers in cervical cancer. *Future Oncol.* 15 3693–3699. 10.2217/fon-2019-0412 31512935

[B52] Wilkerson M. D. Yin X. Hoadley K. A. Liu Y. Hayward M. C. Cabanski C. R. (2010). Lung squamous cell carcinoma mRNA expression subtypes are reproducible, clinically important, and correspond to normal cell types. *Clin. Cancer Res.* 16 4864–4875. 10.1158/1078-0432.CCR-10-0199 20643781 PMC2953768

[B53] Wilkerson M. D. Yin X. Walter V. Zhao N. Cabanski C. R. Hayward M. C. (2012). Differential pathogenesis of lung adenocarcinoma subtypes involving sequence mutations, copy number, chromosomal instability, and methylation. *PloS One* 7:e36530 10.1371/journal.pone.0036530 PMC3349715 22590557

[B54] Xie L. Dang Y. Guo J. Sun X. Xie T. Zhang L. (2019a). High KRT8 expression independently predicts poor prognosis for lung adenocarcinoma patients. *Genes* 10:36. 10.3390/genes10010036 30634629 PMC6360019

[B55] Xie L. Wang Q. Dang Y. Ge L. Sun X. Li N. (2019b). OSkirc: a web tool for identifying prognostic biomarkers in kidney renal clear cell carcinoma. *Future Oncol.* 15 3103–3110. 10.3892/ol.2019.10440 31368353

[B56] Xie L. Wang Q. Nan F. Ge L. Dang Y. (2019c). OSacc: gene expression-based survival analysis web tool for adrenocortical carcinoma. *Cancer Manag Res.* 11 9145–9152. 10.2147/cmar.s215586 31749633 PMC6817837

[B57] Xie Y. Xiao G. Coombes K. R. Behrens C. Solis L. M. Raso G. (2011). Robust gene expression signature from formalin-fixed paraffin-embedded samples predicts prognosis of non-small-cell lung cancer patients. *Clin. Cancer Res.* 17 5705–5714. 10.1158/1078-0432.CCR-11-0196 21742808 PMC3166982

[B58] Yan Z. Wang Q. Sun X. Ban B. Lu Z. Dang Y. (2019). OSbrca: a web server for breast cancer prognostic biomarker investigation with massive data from tens of cohorts. *Front. Oncol.* 9:1349. 10.3389/fonc.2019.01349 31921624 PMC6932997

[B59] Zhang G. Wang Q. Yang M. Yao X. Qi X. An Y. (2020). OSpaad: an online tool to perform survival analysis by integrating gene expression profiling and long-term follow-up data of 1319 pancreatic carcinoma patients. *Mol. Carcinog.* 59 304–310. 10.1002/mc.23154 31912599

[B60] Zhang G. Wang Q. Yang M. Yuan Q. Dang Y. Sun X. (2019). OSblca: a web server for investigating prognostic biomarkers of bladder cancer patients. *Front. Oncol.* 9:466. 10.2217/fon-2019-0296 31275847 PMC6593271

[B61] Zheng H. Zhang G. Zhang L. Wang Q. Li H. Han Y. (2020). Comprehensive review of web servers and bioinformatics tools for cancer prognosis analysis. *Front. Oncol.* 10:68 10.3389/fonc.2020.00068 PMC7013087 32117725

[B62] Zhu C.-Q. Ding K. Strumpf D. Weir B. A. Meyerson M. Pennell N. (2010). Prognostic and predictive gene signature for adjuvant chemotherapy in resected non-small-cell lung cancer. *J.Clin. Oncol.* 28 4417–4424. 10.1200/JCO.2009.26.4325 20823422 PMC2988634

