# Supplementary material for: OSluca: An Interactive Web Server to Evaluate Prognostic Biomarkers for Lung Cancer
Source: Front Genet. 2020 May 26;11:420. doi: 10.3389/fgene.2020.00420 (PMC7264384; doi:10.3389/fgene.2020.00420)
Supplement: Supplementary file 1 [file Data_Sheet_1.DOCX]

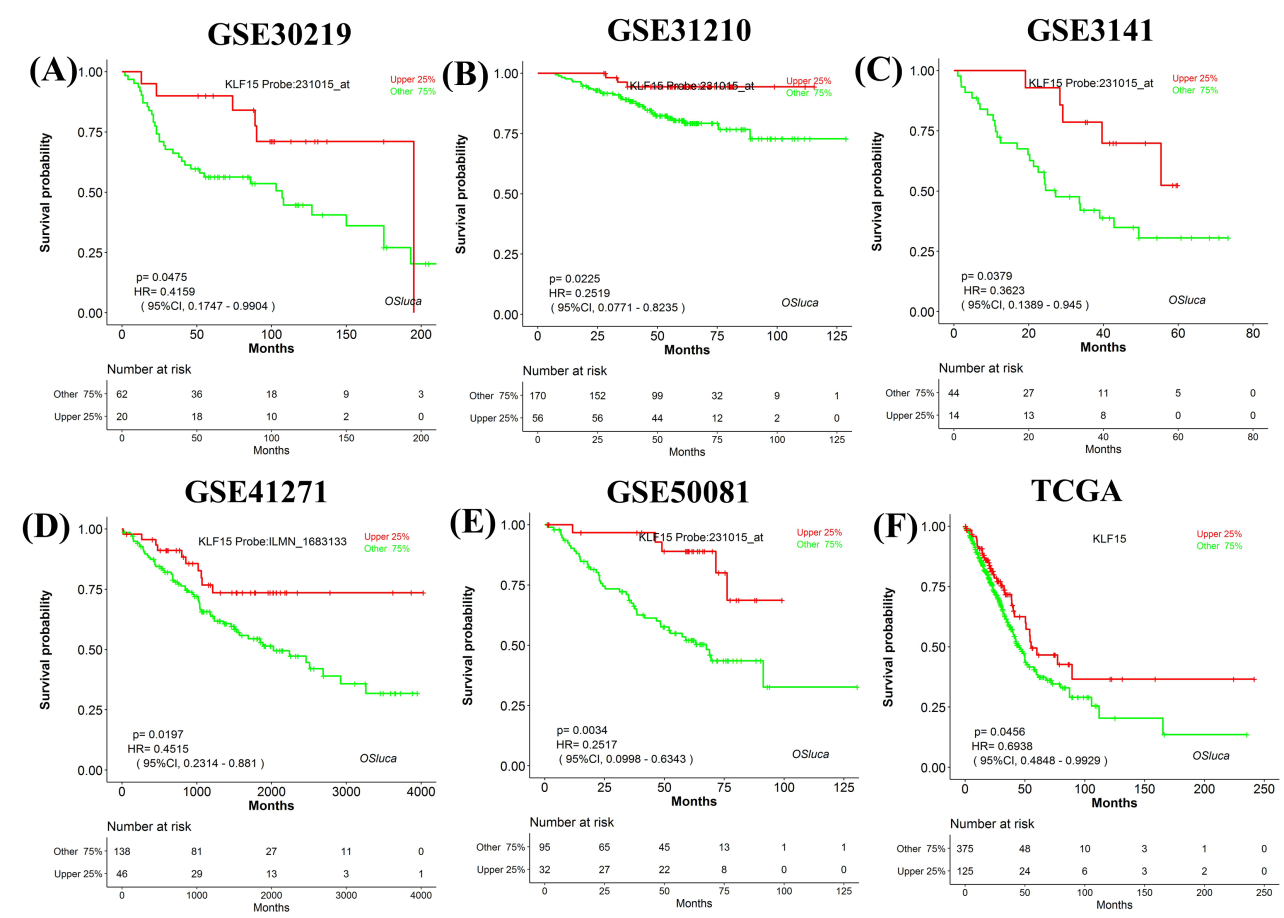


Fig.S1 High expression of *KLF15* gene could be as a good potential prognostic biomarker in lung adenocarcinoma in OSluca, but against the reported research. Gao et al reported that *KLF15* was high expressed on tumor tissue and could be as a poor prognostic biomarker. But in OSluca tool, *KLF15* showed as a good tumor biomarker in lung adenocarcinoma. (A) OS of *KLF15* gene in GSE30219; (B) OS in GSE31210; (C) OS in GSE3141; (D) OS in GSE41271; (E) OS in GSE50081; (F) OS in TCGA in lung adenocarcinoma. The histological type of lung adenocarcinoma was used by those datasets with 25% VS 75% cutoff value. *KLF15*: The Krüppel-like factor 15; OS: overall survival.


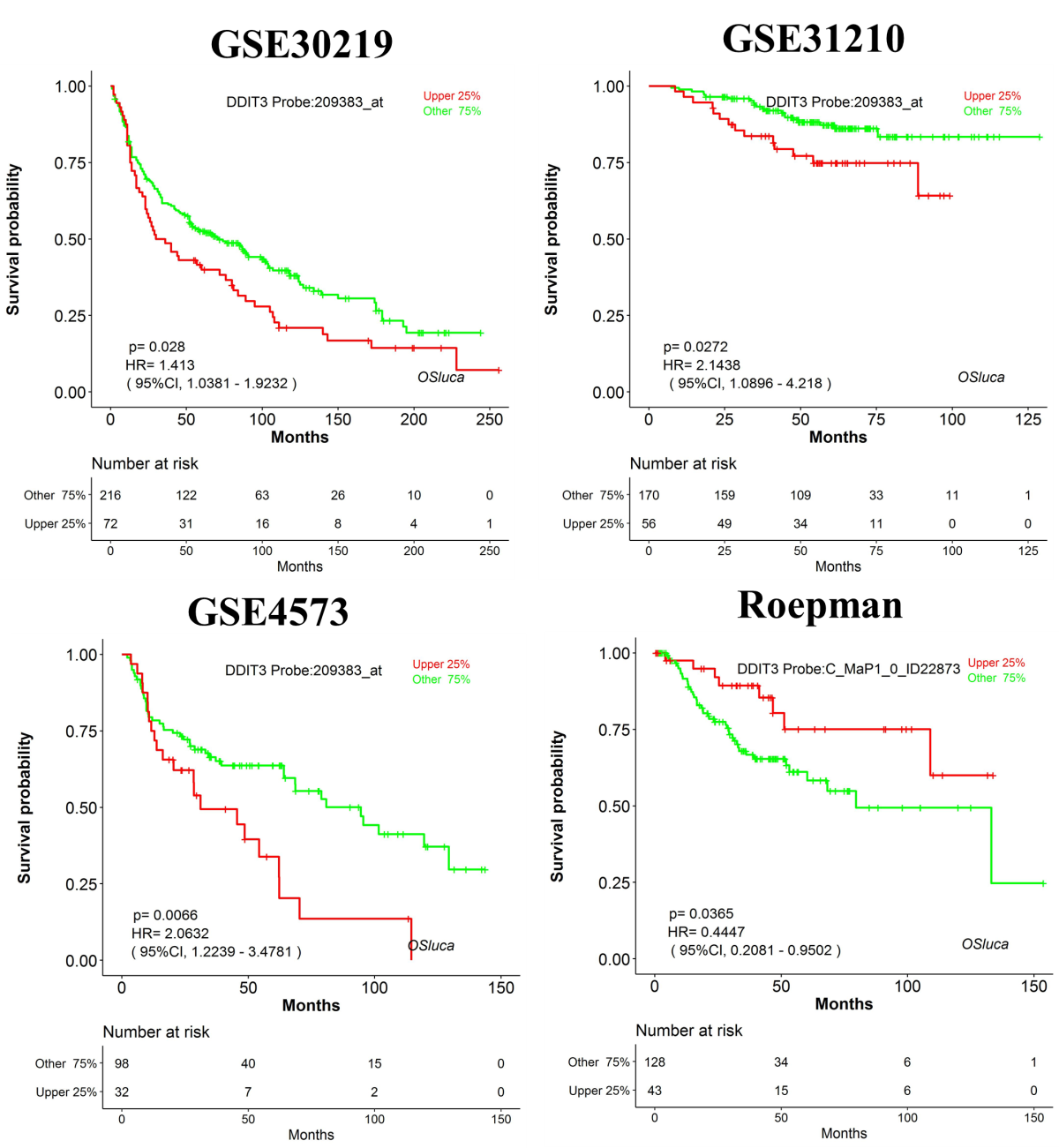


Fig.S2 *DDIT3* gene could be as a dual prognostic potency in lung cancer using independent cohorts by OSluca. (A) Overall survival (OS) of *DDIT3* gene in GSE30219; (B) OS in GSE31210; (C) OS in GSE4573; (D) OS in Roepman dataset; *DDIT3*: DNA-damage-inducible transcript 3, also named C/EBP homologous protein (*CHOP*).
